# In-vivo kinetics of inhaled 5-Aminolevulinic acid-Induced Protoporphyrin IX fluorescence in bronchial tissue

**DOI:** 10.1186/1465-9921-8-33

**Published:** 2007-04-19

**Authors:** Hubert Hautmann, Josef P Pichler, Herbert Stepp, Reinhold Baumgartner, Fernando Gamarra, Rudolf M Huber

**Affiliations:** 1 Medizinische Klinik I, Klinikum rechts der Isar, Technische Universität, D-81675 Munich, Germany; 2 Laser-Forschungslabor an der Urologischen Klinik Großhadern, D-81377 Munich, Germany; 3 Medizinische Klinik-Innenstadt, Klinikum der Ludwig-Maximilians-Universität, D-80336 Munich, Germany

## Abstract

**Background:**

In the diagnosis of early-stage lung cancer photosensitizer-enhanced fluorescence bronchoscopy with inhaled 5-aminolevolinic acid (5-ALA) increases sensitivity when compared to white-light bronchoscopy. This investigation was to evaluate the *in vivo *tissue pharmacokinetics of inhaled 5-ALA within the bronchial mucosa in order to define the time optimum for its application prior to bronchoscopy.

**Methods:**

Patients with known or suspected bronchial carcinoma were randomized to receive 200 mg 5-ALA via inhalation 1, 2, 3, 4 or 6 hours before flexible fluorescence bronchoscopy was performed. Macroscopically suspicious areas as well as areas with visually detected porphyrin fluorescence and normal control sites were measured spectroscopically. Biopsies for histopathology were obtained from suspicious areas as well as from adjacent normal areas.

**Results:**

Fluorescence bronchoscopy performed in 19 patients reveals a sensitivity for malignant and premalignant changes (moderate dysplasia) which is almost twice as high as that of white-light bronchoscopy, whereas specificity is reduced. This is due to false-positive inflammatory lesions which also frequently show increased porphyrin fluorescence. Malignant and premalignant alterations produced fluorescence values that are up to 5 times higher than those of normal tissue. According to the pharmacokinetics of porphyrin fluorescence measured by spectroscopy, the optimum time range for 5-ALA application is 80–270 min prior to fluorescence bronchoscopy, with an optimum at 160 min.

**Conclusion:**

According to our results we propose inhalation of 5-ALA 160 min prior to fluorescence bronchoscopy, suggesting that this time difference provides the best tumor/normal tissue fluorescence ratio.

## Background

The detection of premalignant and early-malignant endobronchial alterations is growing increasingly important in the diagnosis of lung cancer, since an acceptable prognosis is strictly confined to the early stage of the disease [1,2]. However, a simple bronchoscopic method to recognize such alterations is still needed. The yield in localizing very early tumor stages by means of conventional white-light bronchoscopy (WL) alone is poor [3,4]. Therefore, two methods which take advantage of tissue fluorescence have been developed. Autofluorescence (AF) utilizes the difference in light absorption and the concentration of fluorophores in normal and malignant tissues [5,6]. Pharmacologically induced fluorescence can be activated by the inhalation of a photosensitizer. 5-Aminolevulinic acid (5-ALA), a commonly used photosensitizer prodrug, is suitable and safe for endobronchial application [7-9]. Its discriminating ability depends on the cellular uptake of 5-ALA and its subsequent intracellular transformation into protoporphyrin IX (PPIX), the actual fluorescent agent which accumulates in malignant tissue [10,11]. The resulting fluorescence can then be detected bronchoscopically by excitation with violet light and objectified by spectroscopy [12]. *In-vitro *experiments show tumor/normal tissue fluorescence ratios best between 110 and 160 min after exposure to 5-ALA [13]. This study was to evaluate the *in-vivo *tissue pharmacokinetics of inhaled 5-ALA within the bronchial mucosa, in order to define the optimum time range for its application.

## Methods

We recruited patients with known or suspected bronchial carcinoma. To avoid potential drug toxicity, patients with a significant impairment of hepatic or renal function were excluded. The local ethics committee approved the protocol, and a written informed consent was obtained from all patients. 200 mg of 5-ALA (Medac, Hamburg, Germany) dissolved in 5 ml isotonic NaCl, was applied via inhalation with a conventional jet nebulizer (PARI-BOY, Pari, Starnberg, Germany) according to Baumgartner et al. [8]. The patients were randomized to receive 5-ALA 1, 2, 3, 4 or 6 hours before bronchoscopy which was performed under local anesthesia with conventional fiberscopes (11001BC, 11004BC, K. Storz, Tuttlingen, Germany). A sensitive video-camera (Endocam SL-PDD, K. Storz, Tuttlingen, Germany) was connected to the ocular of the bronchoscope and images were displayed on a monitor. The fluorescence mode was used first to search the bronchial system for abnormalities. Macroscopically, porphyrin fluorescence is characterized by a reddish color and can be well identified by visual inspection. For this purpose, an excitation light with wavelengths of 380–440 nm (D-Light, Storz, Tuttlingen, Germany) was applied. Although there are other systems for fluorescence bronchoscopy available (e.g. the LIFE system), the results of trials employing either technology can be directly compared [14].

Spectroscopic measurements were made on various tissue sites, using a sensitive spectrometer (Optical Multichannel Analyser OMA, SI, Penzberg, Germany) which was coupled between bronchoscope and video-camera using a quartz fiber connected to a beam splitter. Porphyrin fluorescence is found at wavelengths greater 630 nm, with a peak emission at 635 nm.

Within areas of positive PPIX-fluorescence the tip of the bronchoscope was directed towards the center of the lesion, and only the central spot was used for spectroscopic analysis. Spectral data were normalized for distance by an application of scattered light at 840 nm, which is reflected from the bronchial tissue. The quantity of porphyrin fluorescence can be calculated by the relation between the intensity of autofluorescence, PPIX fluorescence, and diffuse backscatter at 520, 635 and 840 nm according to the following equation:

[PPIX] ~ [I(635 nm)-0.65*I(520 nm)]/I(840 nm)

PPIX = porphyrin fluorescence [arbitrary units]

I = Intensity [spectroscopically measured value]

Spectroscopy was performed in all macroscopically suspicious areas as well as in areas showing porphyrin fluorescence. Each measurement was repeated three times. In addition, biopsies were obtained from these areas. As a control, adjacent non-suspicious areas were also analyzed spectroscopically and biopsied. The histological results of the biopsies were categorized as "Normal", "Inflammation", "Metaplasia", "Dysplasia Grade I-III (mild, moderate, severe)" or "Malignant". Up to "Mild Dysplasia" the findings were classified as "Benign". All other findings were classified as "(Pre)Malignant". The application-time dependent spectral PPIX values according to equation 1 were fitted for the benign (≤ mild dysplasia) and the (pre)malignant (≥ moderate dysplasia) histologic findings separately. The fit function used was a normal distribution applied to a logarithmic time scale. The two histological ensembles were determined and further analyzed with the Mann-Whitney rank sum test.

## Results

**Figure 1:**
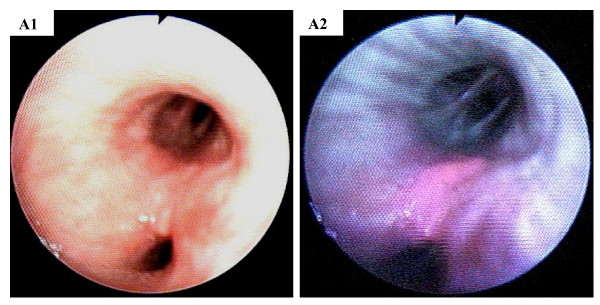
White-light image (A1) and 5-ALA-induced PPIX fluorescence image (A2) of a patient with squamous cell carcinoma.

Nineteen patients were investigated. Basline characteristics of all patients are displayed in table 1. As already demonstrated by Baumgartner et al. [8] no side effects were observed during and after 5-ALA inhalation. Based on the spectroscopic measurements of critical findings (≥ moderate dysplasia) versus normal findings, a method was established to objectify visible color contrasts seen in neoplastic lesions. Figure 1 shows an example for a squamous cell carcinoma with an obvious colour change (red) for the PPIX image. It is difficult to differentiate tumor margins in the white-light mode, even when the tumor appears to be distinctive or exophytic, since there is no detectable color contrast.

**Table 1:** Baseline characteristics of the evaluated patients (n = 16)

**Age**-yr	
Mean	69.0
Range	58 – 86
**Male sex **no. (%)	10 (63)
**Smoker or ex-smoker **no. (%)	14 (88)
**Obstructive lung disease **no. (%)	5 (31)
**Vital capacity **(l)	
Mean	2.72
Range	1.14 – 4.61
**FEV1 **(l)	
Mean	1,85
Range	1.10 – 3.16
**PaO2 **(mmHg)	
Mean	68.1
Range	58.4 – 75.7
**PaCO2 **(mmHg)	
Mean	38.2
Range	33.0 – 43.4

**Figure 2:**
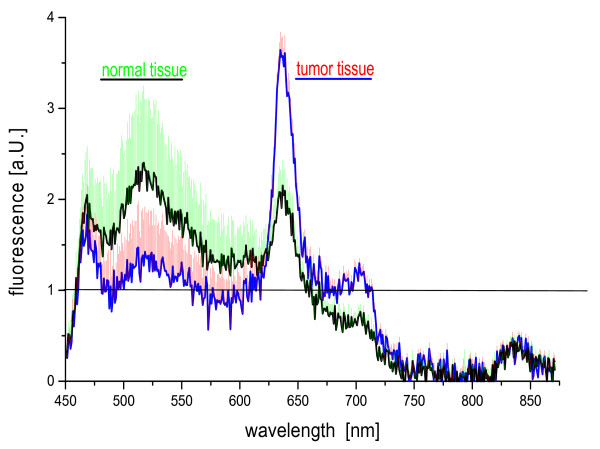
Means and SEM of tumor tissue spectra and normal tissue spectra after inhalation of 200 mg of 5-ALA in the time range from 80–270 min prior to investigation.

Figure 2 illustrates the mean spectral characteristics for tumor and normal tissue after excitation with wavelengths of 380–440 nm. Spectra have been normalized to the remission peak at 840 nm. The spectral quantities of PPIX fluorescence according to equation 1, the visual ratings and the corresponding histological results of each biopsy site are displayed in Table 2. Due to a low signal-to-noise ratio, not all measurements were evaluable. Three patients (Pat# 14+15+16) had to be excluded from analysis, since no valid fluorescence values could be obtained. For this reason, the projected number of patients was eventually extended from 15 to 19.

**Table 2:** Fluorescence values and histological results of all biopsy sites

Pat #	Time after 5-ALA inhalation [min]	Histological result and visual fluorescence			Fluorescence "pathologic tissue" [a.u.] measurement 1–3			Fluorescence "normal tissue" [a.u.] Measurement 1–3		
1	135	Sc +	Sqc +		5.0	3.7	-	2.7	2.6	-
2	245	Sqc +			3.4	-	-	-	-	-
3	75	Sqc +			1.0	-	-	0.5	-	-
4	85	Ade +	Ade +	No +	6.9	3.4	1.6	1.0	0.2	2.2
5	345	Inf +			1.4	-	-	0.5	-	-
6	225	Met +	Hyp +		3.0	6.2	-	1.3	2.8	-
7	135	Hyp +	Hyp -		5.2	2.6	-	1.4	1.1	-
8	60	Met +	Sqc +		2.7	1.4	-	2.1	0.3	-
9	290	No +	Inf +	Inf +	1.2	3.2	1.7	-	1.6	-
10	195	Hyp +	Sqc +		2.5	6.3	-	0.3	1.3	-
11	390	Inf +	Ade +		1.2	1.1	-	-	-	-
12	165	Inf +	Met -	Inf -	1.2	0.7	0.8	0.4	0.8	-
13	140	Hyp -	Inf -	Inf -	1.2	1.3	1.4	-	-	0.9
14	195	Hyp +	Hyp +		-	-	-	-	-	-
15	210	Dys II +	Dys II +		-	-	-	-	-	-
16	180	Sqc +	Inf		-	-	-	-	-	-
17	255	Sqc +	Dys III +		3.3	3.2	-	2.2	2.2	-
18	180	TBC +			3.0	-	-	0.2	-	-
19	210	Sqc +	Sqc +		5.0	3.7	-	2.1	2.8	-

Abbreviations: No = normal, Inf = inflammation, TBC = tuberculosis, Hyp = hyperplasia, Met = metaplasia, Dys = dysplasia grading I-III (mild, moderate, severe), Sqc = squamous carcinoma, Sc = small cell carcinoma, Ade = adenocarcinoma, + = fluorescence positive (visually), - = fluorescence negative (visually), a.u. = arbitrary units. Missing values represent measurements with low signal-to-noise ratio.

**Figure 3:**
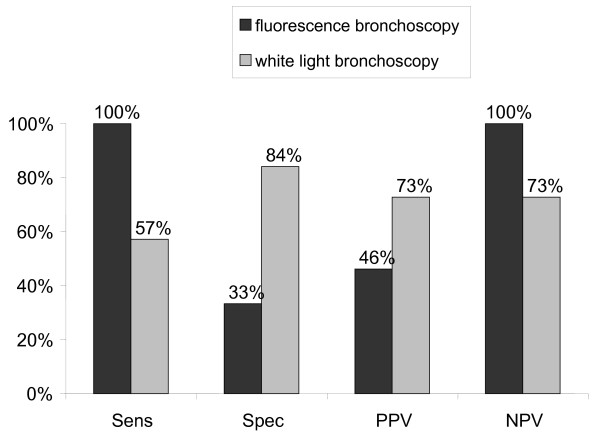
**Sensitivity and specificity of fluorescence bronchoscopy and white-light bronchoscopy in relation to histology results. **n = 19 patients, 38 biopsies; Abbreviations: Sens = Sensitivity, Spec = Specificity, PPV = Positive predictive value, NPV = Negative predictive value

When tumor tissue is compared to normal tissue, a reduced autofluorescence, but a marked increase in PPIX fluorescence becomes evident. Sensitivity, specificity, negative predictive values, and positive predictive values were calculated from the visual ratings of the findings obtained by white-light and fluorescence bronchoscopy in comparison to histology (Figure 3). Fluorescence bronchoscopy reveals a sensitivity which is nearly twice as high as in white-light bronchoscopy. The specificity, however, shows a significant lower level. This is explained by false-positive findings during fluorescence bronchoscopy which were due to the concomitance of inflammatory lesions exhibiting fluorescence values between normal tissue and lesions ≥ moderate dysplasia.

**Figure 4:**
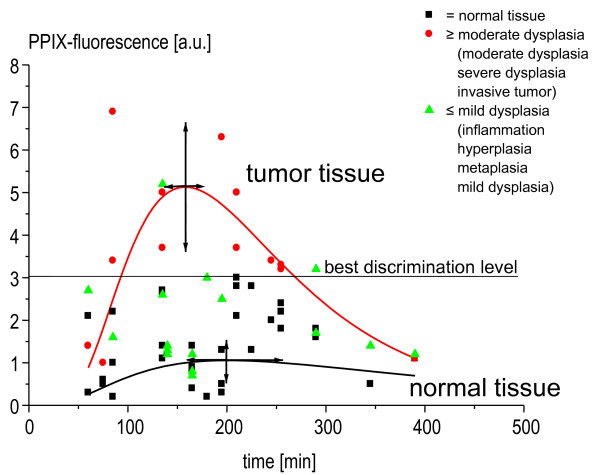
**Values of PPIX-fluorescence in normal findings, findings ≤ mild dysplasia and findings ≥ moderate dysplasia plotted against the time between 5-ALA application and spectroscopy. **Fitted curves are normal distributions on a logarithmic time scale. 19* patients/33 biopsies/86 spectra, * insufficient spectra in 3 patients. Arrows represent the SEM of the maxima of the adopted curves in time and value.

Eventually the calculated fluorescence values were plotted against the time between 5-ALA application and bronchoscopy (Figure 4). It is demonstrated that the different histological classifications produce separate pharmacokinetics. When the curves were fitted to represent normal distributions on a logarithmic time-scale, the maximum fluorescence value for lesions ≥ moderate dysplasia is at 160 min after 5-ALA application. The maximum for normal tissue is at 200 min after 5-ALA application. The spectral values of lesions ≥ moderate dysplasia and of normal tissue differ significantly in the time range of 80 min to 270 min after 5-ALA inhalation (p < 0.01, Mann-Whitney rank sum test). The same accounts for the difference between lesions ≥ moderate dysplasia and lesions ≤ mild dysplaisa. Between the spectra of normal tissue and lesions ≤ mild dysplasia there is no siginificant difference. The mentioned time range is a reasonable period for the detection of 5-ALA-induced PPIX fluorescence, since lesions ≥ moderate dysplasia within this time window exhibit fluorescence values that are 5 times higher (mean value) than those of normal tissue. The PPIX fluorescence values of lesions ≤ mild dysplasia (median 1,55 a.U.) lie between the values of lesions ≥ moderate dysplasia (median 3,4 a.U.) and the values of normal tissue (median 1,3 a.U.).

## Discussion

In contrast to white-light bronchoscopy, pharmacologically induced fluorescence offers certain advantages. The present data provide evidence that the pharmacologically active process of 5-ALA uptake and metabolism produces a higher sensitivity than white-light bronchoscopy alone. However, this advantage is partly compensated by a reduced specificity, since e.g. some areas of inflammation or metaplasia can generate false-positive results. In this context the issue of "per lesion analysis" has to be taken into consideration since it may represent a potential flaw in the statistical evaluation concerning sensitivity, specificity and predictive values, as impressively demonstrated by Chang et al. [15]. As only two sites (one positive area and one control) were biopsied in most of the study subjects our results, however, represent more a "per subject analysis" than a "per lesion analysis".

According to *in-vitro *studies with co-cultures, best fluorescence intensities were to be expected between 110 and 160 min after inhalation of 5-ALA [13]. Our results favor the performance of fluorescence bronchoscopy within a time period between 80 and 270 min after the inhalation of 5-ALA, with a calculated maximum of fluorescence intensity at 160 min. In order to seize the highest possible discrimination between normal and pathologic tissue, we therefore recommend the application of 5-ALA 160 min before fluorescence bronchoscopy is performed.

The observed heterogeneity of 5-ALA-induced fluorescence intensity in premalignant and malignant changes may be a distinctive feature of 5-ALA metabolism as well as the patterns of tumor invasion. This was also found in experiments with co-cultures, even after correction for tumor cell density [16]. Correlations between the baseline characteristics of the patients and fluorescence values were not detected. Thus, it remains unclear, whether smoking status or lung function excert influence on 5-ALA metabolism. Despite this heterogeneity, the fluctuations in our spectroscopic measurements are still small enough to allow discrimination between harmless and severe findings, with fluorescence values differing by a factor of five (Figure 4). In this context, the adoption of a normal distribution on a logarithmic time-scale was superior to a three compartment model. It delivers the time and the intensity of the calculated peak fluorescence values with discriminating differences between normal and pathologic findings. As reported in other studies [6,9,17], there is always an increase in sensitivity and a decrease in specificity when, for the detection of (pre)malignant changes, white-light bronchoscopy is combined with ALA-enhanced fluorescence bronchoscopy.

## Conclusion

5-ALA-supported fluorescence bronchoscopy enables an increased sensitivity in the bronchoscopic detection of endobronchial malignant and premalignant changes. The clinical implication of this method is the possibility to discover very early-stage lung cancer, in order to markedly improve healing rates and prognosis. With 160 min we propose an optimized time-point in the application of 5-ALA prior to the performance of fluorescence bronchoscopy. In this context, this study can contribute importantly to the efficiency of fluorescence bronchoscopy, particularly with regard to the in-vivo kinetics of 5-ALA. Clinical trials, however, will have to evaluate the significance and the clinical relevance of this method. Of particular interest will be the comparison with autofluorescence bronchoscopy and, especially, whether the addition of inhaled 5-ALA can further improve this technique, since a large multicenter trial has recently shown a benefit for autofluorescence bronchoscopy over white light bronchoscopy [18].

## Competing interests

The author(s) declare that they have no competing interests.

## Authors' contributions

HH carried out the bronchoscopic examinations, participated in spectroscopy and drafted the manuscript. JP carried out all spectroscopic measurements, took part in writing the manuscript and performed the statistical analysis. HS, RB, FG and RMH conceived of the study, and participated in its design and coordination. All authors read and approved the final manuscript.
